# FTO modulates fibrogenic responses in obstructive nephropathy

**DOI:** 10.1038/srep18874

**Published:** 2016-01-04

**Authors:** Chao-Yung Wang, Shian-Sen Shie, Ming-Lung Tsai, Chia-Hung Yang, Kuo-Chun Hung, Chun-Chieh Wang, I-Chang Hsieh, Ming-Shien Wen

**Affiliations:** 1 Department of Cardiology, Chang Gung Memorial Hospital, and Chang Gung University College of Medicine, Taiwan; 2 Department of Infectious Diseases, Chang Gung Memorial Hospital and Chang Gung University College of Medicine, Taiwan

## Abstract

Genome-wide association studies have shown that variants in fat mass and obesity-associated (*FTO*) gene are robustly associated with body mass index and obesity. These *FTO* variants are also associated with end stage renal disease and all-cause mortality in chronic kidney diseases. However, the exact role of FTO in kidneys is currently unknown. Here we show that FTO expression is increased after ureteral obstruction and renal fibrosis. Deficiency of the *FTO* gene attenuates the fibrogenic responses induced by ureteral obstruction in the kidney. Renal tubular cells deficient of *FTO* produce less α-SMA after TGF-β stimulation. FTO is indispensable for the extracellular matrix synthesis after ureteral obstruction in kidneys. Indeed, global gene transcriptions amplitude is reduced in FTO deficient kidneys after ureteral obstruction. These data establish the importance of FTO in renal fibrosis, which may have potential therapeutic implications.

Genome-wide association studies have shown that variations in the first intron of the *Fat mass and obesity associated* (*FTO*) gene are associated with obesity and diabetes in global studies of different ethnicities^1^. The variation at *FTO* rs7202116 locus is shown to be associated with phenotypic variability in body mass index^2^. However, FTO has multiple functions other than associations with obesity. FTO transgenic mice displayed increased obesity^3^ and *FTO* deficient mice have increased prenatal mortality with multiple organ abnormalities^4^. Humans with *FTO* gene loss-of-function mutations also have facial dysmorphism, brain abnormalities, and heart diseases^5^. Recent findings that those *FTO* variants are associated with *IRX3* further imply that FTO could have important functions other than controlling body weight or metabolism^6^.

FTO is a 2-oxoglutarate-dependent *N*^6^-methyladenosine RNA (m^6^A) demethylase^7^. FTO is expressed in most tissues and is abundant in the hippocampus, cerebellum, and hypothalamus^8^,^9^. Clinical studies have implied that FTO functions are linked to lipolysis^10^, telomere length^11^, food intake^12^, breast cancer^13^, and Alzheimer’s disease^14^. FTO levels are regulated by essential aminoacids and FTO-deficient cells have increased autophagy and reduced mammalian target of rapamycin signaling^15^. Furthermore, FTO regulates dopaminergic signaling in midbrain^16^, interacts with CaMKII/CREB pathway^17^, associates with ciliopathies through Wnt signaling^18^, and involves in leptin receptor/STAT3 in brain^19^. Moreover, FTO affects circadian rhythm through inhibiting the CLOCK-BMAL1-induced transcription^20^. These studies provide complex evidences of fundamental FTO functions in many organs or cells. However, the function of FTO in kidneys is still unknown.

Previous case-control clinical studies have suggested the associations between *FTO* polymorphism and risks of end-stage renal disease (ESRD). The *FTO* rs17817449 variants are associated with increased risks of chronic kidney diseases (CKD) and onset of ESRD^21^. The *FTO* polymorphism is also an independent predictor of all-cause mortality in patients with CKD. Meta-analysis for 1540 CKD patients cohorts showed that individuals with the A allele in rs708259 polymorphism on intron 8 of *FTO* had a 42% excess risk of death^22^. The underlying mechanisms of these associations and the role of FTO in kidneys are currently unknown. We hypothesize that FTO plays important role in kidneys and regulates kidney fibrogenic response, a critical underlying mechanism of CKD.

## Results

### FTO levels in kidneys are increased after ureteral obstruction

**Figure 1:**
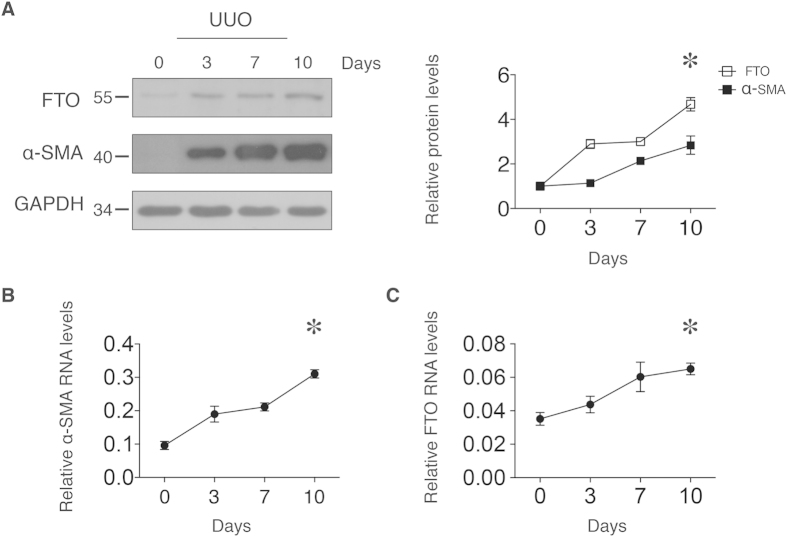
FTO levels in mouse kidney after ureteral obstruction. Mice kidneys were sampled from 0 to 10 days after UUO and analyzed for mRNA and protein concentrations. (**A**) Representative western blot images and densitometry quantification of FTO and α-SMA protein levels from mice kidneys. (**B**) Kidney *α-SMA* and (**C**) *FTO* mRNAs were analyzed and normalized to 18S. Data represent means ± SD, *n* = 3 mice per time point. *P < 0.05 by one-way ANOVA (effect of time).

To investigate the role of FTO in kidneys, we first analyzed the FTO protein abundance and mRNA expression levels in the kidneys after unilateral ureteral obstruction (UUO). We first confirmed that there were significant kidney fibrosis and increases of mRNA and protein of alpha-smooth muscle actin (α–SMA) after UUO surgery (Fig. 1A,B). The α–SMA is the actin isoform characteristic of vascular smooth-muscle cells^23^ and a marker for kidney fibrosis^24^,^25^. After UUO, the FTO protein concentrations increased by 4.78-fold in the kidneys from day 3 to day 10 (Fig. 1A). The mRNA levels of *FTO* increased from day 3 to day 10 (Fig. 1C). The increases of FTO after UUO implied FTO plays certain role in the kidney. Based on this observation, we hypothesized that FTO regulates tubulointerstitial fibrosis after UUO in the kidney.

### Tubulointerstitial Fibrosis is decreased in FTO deficient mice

**Figure 2:**
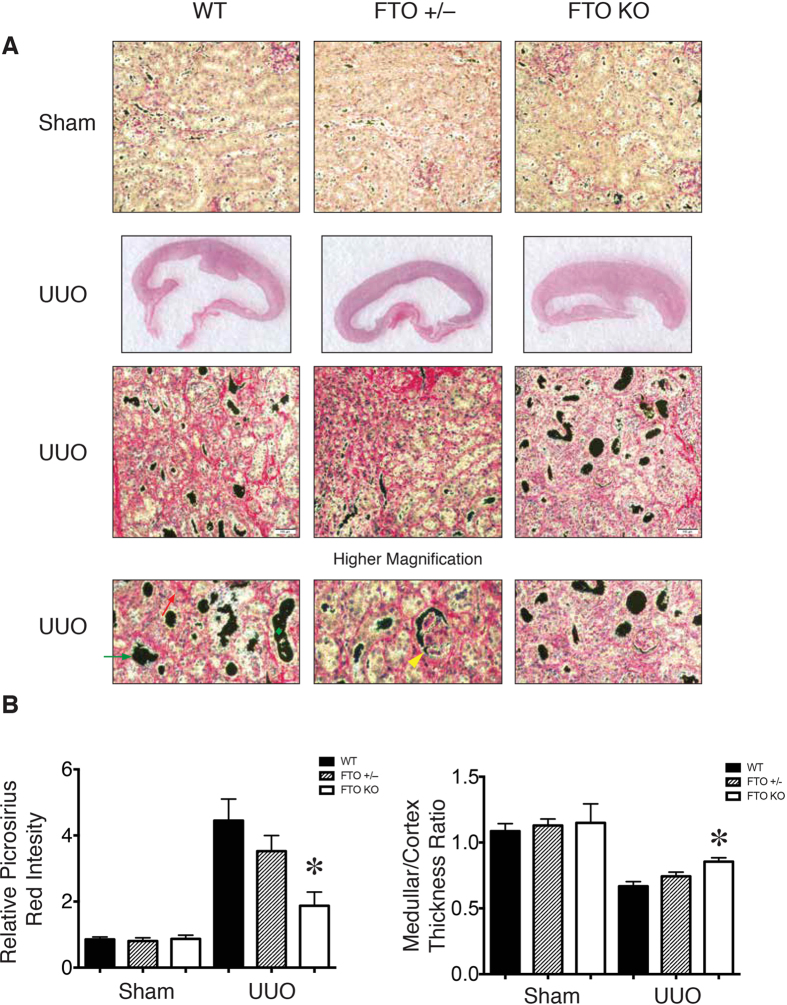
FTO deficiency attenuated renal fibrosis and extracellular matrix deposition. (**A**) Representative picrosirius red staining and gross pathology images of wild-type, *FTO*^+/–^, and *FTO*^–/–^ kidney sections for assessment of total collagen deposition and extent of fibrosis. Higher magnification images compared tubular dilation (Green diamond), flattened tubular epithelial cells (Green arrow), focal glomerular sclerosis (Yellow arrowhead), and extracellular matrix deposition (Red arrow) in the cortex of obstructed kidneys. (**B**) Quantitative analysis of collagen deposition and medullary/cortex thickness ratio in wild-type, *FTO*^+/–^, and *FTO*^–/–^ mice. 10–12 weeks female wild-type (*n* = 10), *FTO*^+/–^ (*n* = 10), and *FTO*^–/–^ (*n* = 6) mice with body weight of 25.0±1.3, 24.3±1.1, and 21±1.5 g, respectively. Data represent means ± SD. *P < 0.05 by two-sample Mann-Whitney analysis, WT compared to *FTO*^–/–^ mice.

To study whether FTO indeed has critical role in tubulointerstitial fibrosis in the kidney, we performed UUO surgery in wild-type, *FTO*^+/–^, and *FTO*^–/–^ mice and analyzed the severity of tubulointerstitial fibrosis at day 10. After UUO surgery, wild-type mice had renal tubulointerstitial fibrosis, tubular dilation, glomerular sclerosis, and flattened tubular epithelial cells as evidenced by picrosirius red staining (Fig. 2A). In comparison with wild-type kidneys, the *FTO*^–/–^ kidneys exhibited less tubulointerstitial damage, better medullar/cortex thickness ratio, and less fibrosis after UUO surgery (Fig. 2A,B). This result indicated that FTO deficiency protected kidneys from UUO injury.

**Figure 3:**
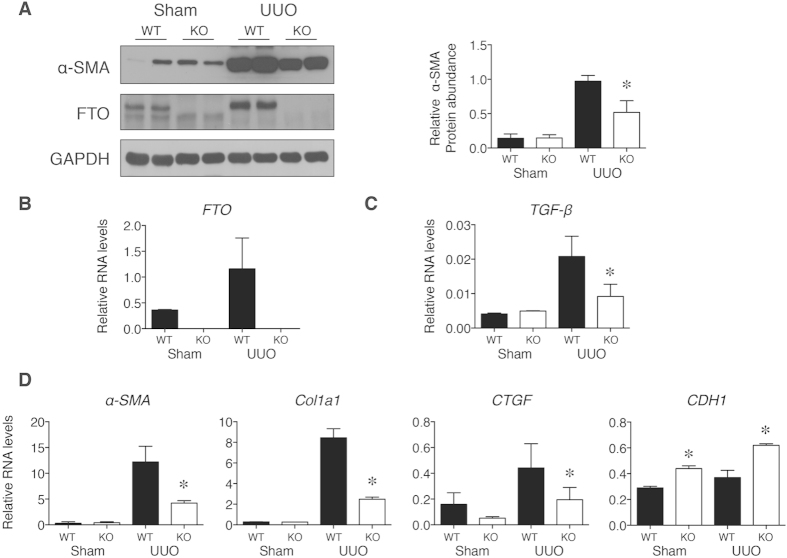
Protein and RNA levels in WT and FTO deficient mice (KO) after UUO. (**A**) Representative western blot images and densitometry quantification of α-SMA protein concentrations from mice kidneys. (**B–D**) Kidney mRNAs levels after UUO surgery were analyzed and normalized to 18S. Data represent means ± SD, *n* = 6 mice per time point. *P < 0.05 by two-sample Mann-Whitney analysis, WT UUO groups compared to KO UUO groups.

After UUO surgery, the α-SMA and FTO protein concentrations increased in wild-type kidneys (Fig. 3A,B). *FTO*^–/–^ kidneys had significantly lower α-SMA protein concentrations after UUO surgery when compared with that in wild-type kidneys (Fig. 3A). The *transforming growth factor-β* (*TGF-β*) mRNA levels were significantly lower in *FTO*^–/–^ kidneys after UUO compared with that in wild-type kidneys (Fig. 3C). Furthermore, the *a-SMA, collagen, type I, alpha 1* (*Col1a1*), and *connective tissue growth factor* (*CTGF*) mRNA, which were markers for kidney fibrosis and downstream targets of TGF-β^26^,^27^, also exhibited significantly lower levels in *FTO*^–/–^ kidneys after UUO (Fig. 3D). *CDH1* (E-cadherin) is a downstream factor of TGF-β and serves as a marker for epithelial to mesenchymal transition (EMT) of kidney proximal tubular cells^28^. Initiation of EMT is associated with reduced expression of *CDH1*^29^. After UUO surgery in *FTO*^–/–^ mice, *CDH1* mRNA levels were higher and implied a decreased EMT response with FTO deficiency (Fig. 3D). Taken together, these observations suggest that FTO plays an important role in fibrogenic response obstructive nephropathy.

### Deficiency of FTO inhibits TGF-β stimulated α-SMA protein expression

**Figure 4:**
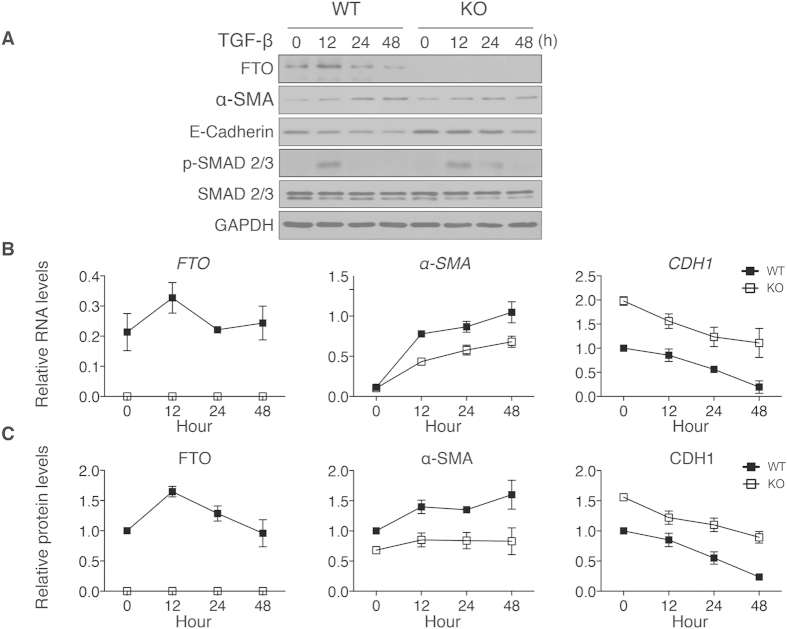
FTO deficiency attenuated α-SMA concentrations induced by TGF-β in renal tubular cells. (**A**) Representative western blot images of FTO, α-SMA, E-Cadherin, phospho-SMAD 2/3, and SMAD 2/3 protein concentrations after TGF-β (1ng/mL) stimulation in wild-type and *FTO*^–/–^ renal tubular cells. (**B**) Quantitative analysis of *FTO, α-SMA*, and *CDH1* (*E-Cadherin*) mRNA levels in wild-type and *FTO*^–/–^ renal tubular cells after TGF-β stimulation (*n* = 6). (**C**) Quantitative analysis of FTO, α-SMA, and CDH1 (E-Cadherin) protein concentrations in wild-type and *FTO*^–/–^ renal tubular cells after TGF-β stimulation (*n* = 3). Data represent means ± SD. *P < 0.05 by two-way ANOVA.

Previous studies have shown that TGF-β signaling is a key mediator in renal fibrosis after UUO^30^. *FTO*^–/–^ kidneys had lower *TGF-β* expressions after UUO (Fig. 3C). We thought to examine whether deficiency of FTO inhibits TGF-β stimulation of *α-SMA* expression and other downstream signaling factors in isolated renal tubular cells. After TGF-β stimulation, *FTO* mRNA levels increased at 12 h and returned to baseline at 24  while FTO protein concentrations increased from 12 to 24 h and returned to baseline at 48h (Fig. 4A–C). The *α-SMA* mRNA levels and protein concentrations increased at 12, 24, and 48 h in wild-type renal tubular cells after TGF-β stimulations, (Fig. 4A–C). FTO deficient renal cells exhibited significant lower *α-SMA* mRNA and protein concentrations when compared with wild-type cells after stimulation (Fig. 4A–C). Consistent with the findings in UUO kidneys, *CDH1* mRNA levels and protein concentrations were significantly higher in FTO deficient renal cells (Fig. 4A–C). There were no significant differences of phospho-SMAD 2/3 and SMAD 2/3 protein abundance between FTO deficient and wild-type cells (Fig. 4A). These data supported that FTO deficiency affects the downstream factors of TGF-β signaling, such as α-SMA and CDH1.

### FTO modulates UUO-dependent gene transcriptions

**Figure 5:**
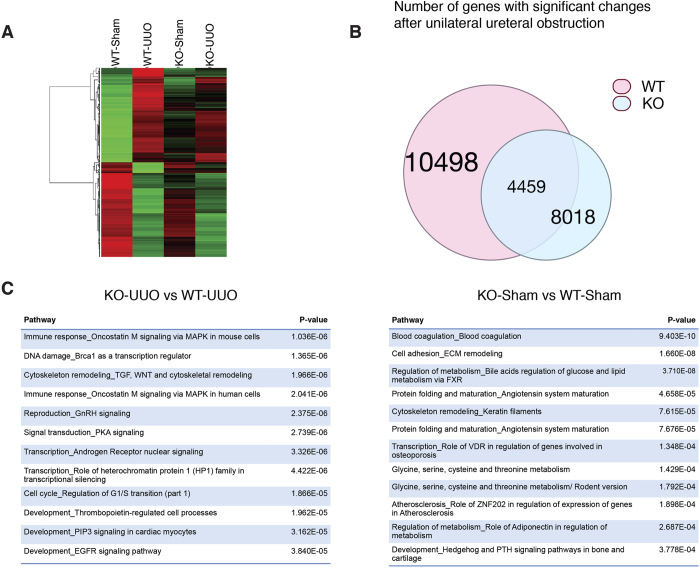
FTO modulates gene transcriptions after UUO. (**A**) Heat map: color denoted UUO-induced top 200 increases or decreases in gene transcripts from WT or FTO deficient mice (KO). (**B**) Significant transcripts after UUO in wild-type (pink circle) or FTO deficient (blue circle) kidneys. (**C**) Pathway analysis between KO and WT mice after UUO or sham procedure by MetaCore.

FTO levels affect RNA modification and transcriptome^31^. It can act as transcription co-activator and affect transcription processes^20^,^32^. Based on the findings that tubulointerstitial fibrosis and TGF-β was attenuated in *FTO* deficiency mice, we reasoned that FTO was critical in mediating global gene transcriptions after UUO surgery. To determine whether FTO modulates endogenous gene transcriptions after UUO, we analyzed the global gene expression changes of wild-type and *FTO*^–/–^ mice after UUO (Fig. 5A). UUO are expected to result in both positive and negative transcriptional responses in kidneys^33^. If deficiency of FTO is independently capable of abrogating transcriptions by UUO, we would expect to see a global repressive shift in the transcriptional response to UUO. Indeed, we found that the transcriptional response after UUO in FTO deficient mice had significantly less amplitude when compared to wild-type mice. When comparing the top 200 activated or repressed genes in wild-type mice, genes in FTO deficient mice exhibited less activation or repression (Fig. 5B). FTO deficient mice had 1412 less genes activated and 1068 less genes repressed compared to wild-type mice. We analyzed the differential expressed genes between wild-type and FTO deficient mice with MetaCore package. Comparing between wild-type and FTO deficient mice after UUO or sham procedure, top 12 gene pathway maps affected by FTO were summarized in Fig. 5C. The immune response/MAPK, cytoskeleton/TGF, DNA damage/BRCA1, and blood coagulation pathway were the top ranked pathway affected by FTO deficiency after UUO. Taken together, these data concluded that FTO deficiency decreases fibrogenic responses and protects kidney from UUO associated fibrotic damages.

## Discussion

Our data provide a mechanistical insight into the association of FTO and chronic kidney diseases. FTO expression levels were altered after UUO and deficiency of FTO results in decreased fibrogenic responses. FTO deficiency resulted in decreased α-SMA synthesis in renal tubular epithelial cells after TGF-β stimulation. Indeed, global gene transcriptions amplitude was reduced in FTO deficient mice. Pathway analysis revealed that deficiency of FTO affects immune response, DNA damage, and cytoskeleton remodeling through TGF-β signaling.

This important role of FTO in kidneys implies a link between obesity and kidney. Overweight patients without diabetes or hypertension had increased risk for CKD^34^. However, in patients with ESRD, a higher body mass index is paradoxically associated with better survival^35^. The exact relationships between obesity and CKD are still unclear. Our results provide a direct link between obesity gene variant and kidney fibrogenic responses, which could be a possible key for future therapeutic choice. Although recent data suggested that *Irx3* is a functional target of variants within introns of *FTO*^36^, the exact role of FTO or IRX3 in human obesity is still unclear^37^. The role of IRX3 in kidneys also remains unknown. Further studies will be needed to dissect the roles of FTO and IRX3 in kidney and CKD patients.

Our data showed that several downstream targets of TGF-β, such as *α-SMA* or *CTGF*, had decreased responses to stimulation by TGF-β or UUO with FTO deficiency. These results implied that FTO not only affected TGF-β levels (Fig. 3C) but also acted downstream of TGF-β. Previous studies have shown that FTO is able to act as a transcription co-activator^17^,^20^ and has a role in RNA methylation^38^. Our results showed that phosphorylation and protein abundance of SMAD, which transduce extracellular TGF-β signals to nucleus, were unaffected by FTO deficiency. These data indicated that FTO may act downstream of SMAD to affect TGF-β targets. Future study will need to investigate the exact mechanism how FTO modulates these TGF-β regulated genes. Moreover, our pathway analysis showed that FTO also affected immune response, DNA damage, and cytoskeleton remodeling pathways. It is also possible that FTO exerts broad influences upon several signaling pathways to affect kidney fibrosis besides TGF-β signaling.

FTO is implicated in several signaling pathways, including mTOR, CREB, Wnt, and STAT3^1^. Our results proved that FTO also plays an important role in TGF-β signaling. Previous studies have shown that TGF-β and obesity are closely related^39^,^40^,^41^. Hypothalamic TGF-β is overproduced by astrocytes and proopiomelanocortin neurons under conditions such as obesity and aging^40^. It is then reasonable that deficiency of FTO also affects TGF-β signaling. TGF-β regulates multiple cellular functions including survival, proliferation, differentiation, and migration^42^. In kidneys, TGF-β governs a variety of pathophysiological function, such as inflammation, fibrogenesis, epithelial-to-mesenchymal transition, and metabolism^30^. Our observations that FTO modulates the fibrogenic response in kidneys and TGF-β signaling open several speculations whether FTO deficiency also affects epithelial-to-mesenchymal transition or inflammatory responses in the kidney. Future studies will answer these questions.

## Materials and Methods

### Cell culture and Antibody

Mouse proximal tubular epithelial cells were isolated from wild-type or *FTO*^–/–^ mice. The kidneys were de-capsulated and the medulla removed. The cortices were finely dissected and digested with collagenase type-II. The kidney digests were filtered with 70μm sieve (BD) and cell pellets were resuspended in renal cell culture medium (DMEM-F12, 10% FBS, 5 μg/mL insulin, 5 μg/mL transferrin, 50 nM selenium, 5 nM T3, 50 mM hydrocortisone, and Penicillin/Streptomycin). Helper-dependent adenovectors (Microbix) were generated with mouse *FTO* cDNA in the shuttle vector pDC516 and Flp-FRT system. Antibodies used for immunoblotting and immunofluorescence included anti-FTO (Abnova, PAB11419), α-SMA (Sigma), GAPDH (Cell signaling), phosphor-SMAD 2/3 (Cell signaling), and SMAD2/3 (Cell signaling).

### Animals and surgery

All animal experimental protocols were approved by Chang Gung University and Chang Gung Memorial Hospital Institutional Animal Care and Use Committee. All experiments were performed in accordance with the approved protocols, guidelines, and regulations.

Embryonic stem cells and mice with loxP sites surrounding FTO exon 3 were obtained from EUCOMM (Institute of Developmental Genetics) and Mouse Genetics Programme (Wellcome Trust Sanger Institute). FTO-deficient mice were generated from matings between FTO^*flox/flox*^ mice and Ella-cre mice (Jackson Laboratory). Homozygous FTO-deficient, heterozygous FTO-deficient, and wild-type mice were generated from matings between two heterozygous mice. The unilateral ureteral obstruction procedure was performed under general anesthesia with ketamine and xylazine (80/6 μg/g intraperitoneally). The incision was from left flank area with scalpel. Ureters were then explored and ligated with 2-0 silk with double ligature. After ligation, the operative fields were rinsed with sterile PBS to prevent future adhesion. After 3 or 7 or 10 days, mice were sacrificed and kidneys were harvested for further analysis.

### Protein and mRNA analysis

Total RNA was extracted using TRI reagent (Ambion) according to the manufacturer’s instructions. One microgram of total RNA was reverse-transcribed and analyzed using the Applied Biosystems Real-time PCR system. The relative gene expression method was used for analysis, and the expression of the target genes was normalized to that of 18S rRNA. The assay was repeated independently at least three times. Protein was isolated from homogenized frozen kidneys or cells with cell lysis buffer (Cell Signaling). The lysates were separated by electrophoresis, transferred to polyvinylidene fluoride membranes, and probed with specific antibodies. The results were normalized to GAPDH band and calculated with Image J (NIH).

### Immunohistochemistry analysis

The kidney after UUO or sham operations were fixed with 4% PFA and processed for paraffin embedding. The sections were then deparaffinize/rehydrated and stained with or without Weigerts Hematoxylin. The staining was then proceeded with modified picrosirius staining kit (Polysciences, #24901) according to manufacturing protocol. The results were analyzed with Adobe Photoshop CS2 and ImageJ (NIH).

### Microarray analysis

Microarray experiments were performed at Genomic Medicine Research Core Laboratory (GMRCL) of Chang Gung Memorial Hospital. The RNA samples from kidneys of wild-type and *FTO*^–/–^ mice 10 days after sham or UUO surgery were hybridized using Affymetrix Mouse Genome 430A 2.0 Oligonucleotide Microarrays. All analysis was done in duplicate and dye swap experiments were used. The signals that were differentially expressed >2 or <0.75 were considered significant and further analyzed. Network and pathway analyses were performed with MetaCore (GeneCo).

### Statistical analysis

Values were expressed as mean ± standard deviation. Data were compared using Student’s t-tests or analysis of variance (ANOVA), where appropriate. For data with small numbers and non-normal distribution, two-sample Mann-Whitney analysis was used. *P* < 0.05 was considered statistically significant.

## Additional Information

**How to cite this article**: Wang, C.-Y. *et al.* FTO modulates fibrogenic responses in obstructive nephropathy. *Sci. Rep.* **6**, 18874; doi: 10.1038/srep18874 (2016).
